# Where to look for power Laws in urban road networks?

**DOI:** 10.1007/s41109-018-0060-9

**Published:** 2018-04-04

**Authors:** Meisam Akbarzadeh, Soroush Memarmontazerin, Sheida Soleimani

**Affiliations:** 1 Department of Transportation Engineering, Isfahan University of Technology, Isfahan, Iran; 2 Department of Civil Engineering, University of Isfahan, Isfahan, Iran

**Keywords:** Urban road networks, Centrality, Power law, Vulnerability

## Abstract

Spatial embeddedness and planarity of urban road networks limit the range of their node degree values. Therefore, pursuing analysis based on the distribution of node degrees e.g. scale free aspect could not be accomplished in urban road networks. We have inspected the distribution of degree, betweenness centrality, weighted degree (based on incident link capacities), and alpha weighted degree for eight urban road networks across the world. These networks are abstracted from Philadelphia (USA), Berlin (Germany), Chicago (USA), Anaheim (USA), Gold Coast (Australia), Birmingham (UK), and Isfahan (Iran). Our results show that although the degree (weighted and unweighted) distributions of these networks are totally different, they all show power law distributions in betweenness centrality. Thus, scale free aspect could be observed in the betweenness centrality distribution. We then analyzed the collapse of network as a result of node removals. The collapse patterns suggest that critical nodes of urban road networks could not be detected solely based on betweenness centrality. Therefore, we conclude that the concept of betweenness centrality in urban road networks is more of functional merit than topological merit. In other words, central nodes play an important role in transmitting the flow but their loss would not harm the connectivity of urban networks. This claim is supported by analyzing the correlation among node flow and node betweenness in Isfahan and Anaheim.

## Introduction and methodology

In an urban context, by abstracting intersections as nodes and streets as links, an urban road network is developed. Urban road networks have certain differences with some other critical infrastructures. These differences are mainly due to two aspects of urban road networks. Firstly, urban road networks are spatially embedded and planar. Moreover, these are finite networks, and width of the roads the links represent are non-vanishing. Therefore, the range of variation of their node degrees is limited. Secondly, the nature of route selection and the effect of congestion on link traversal cost are not the same as other infrastructure networks such as power and data transmission networks. In urban road networks, every traveler aims to select the shortest path to travel from her origin to destination. On the other hand, the travel time of each link depends on the number of travelers moving on it. Hence, every traveler selects her path according to the prevailing conditions of the network flow and also affect the prevailing conditions of the network once she starts her trip. In many non-transportation networks, the link travel times are independent of their loads. These aspects of urban road networks make the application of many concepts and methods of the complex network literature cumbersome. For instance, the degree of an urban network node is an integer within the range of 1 through 12. Values of 1 and 12 are barely observed. Therefore, fitting a power-law distribution or a log-log equation of node degrees would be statistically impossible due to the insufficiency of observations (by “observation” we mean the realization of node degrees. One gets at most only 12 observations which is not sufficient for fitting a regression with two parameters to estimate). This implies that the concept of scale free networks could not be easily pursued in urban road networks.

In order to overcome obstacles caused by the limited range of node degree values, we analyze other network metrics and see if global patterns could be detected in them across different urban contexts. We abstracted the network by assuming intersections as nodes and streets as links.

Many indices have been developed to quantify the centrality of network nodes. These indices could be classified into structural and iterative refinement centralities (Lü et al. 2016). Structural centralities in turn, may be categorized into neighborhood-based and path-based. Neighborhood-based centralities including degree, LocalRank (Chen et al. 2012), ClusterRank (Petermann and De los Rios 2004), coreness (Kitsak et al. 2010), H-Index (Hirsch 2005), etc., focus on the number and influence of the nodes connected to each node. On the other hand, path-based centralities including eccentricity (Hage and Harary 1995), Katz centrality (Katz 1953), information index (Stephenson and Zelen 1989), betweenness centrality (Bavelas 1948), subgraph centrality (Estrada and Rodriguez-Velazquez 2005), etc. consider the position of a node in the network and the paths which the node is part of. Iterative refinement centralities include eigenvector centrality (Bonacich and Lloyd 2001), PageRank (Brin and Page 2012), LeaderRank (Lü et al. 2011), HITs (Kleinberg 1999), SALSA (stochastic approach for link structure analysis) (Lempel and Moran 2000), etc.

Opsahl et al. (2010) developed Alpha weighted degree which considers both degree and the weights of links to assess the centrality of a node. Kazerani and Winter (2009a) and Kazerani and Winter (2009b) developed modified betweenness centrality measures to predict and explain traffic flow in transportation networks.

Centrality measures are used to rank the nodes of networks according to their importance (Wasserman and Faust 1994; Scott and Carrington 2011). We calculated the values of four major measures and analyzed them for eight different cities around the world. These four centrality measures include degree, betweenness centrality, weighted degree based on link capacities, and alpha degree. Hence, we have analyzed two neighborhood-based, one path-based, and one combined index.

Degree (*d*_*i*_) represents the number of neighbors of node *i*. Betweenness Centrality (*C*_*B*_(*i*)) “measures the extent to which a node lies on the shortest paths between any pair of nodes” (Newman 2010). The betweenness centrality of node *i* (C_B_(i)) is formulated as:

1 $$ {C}_B(i)=\sum \limits_{s\ne t}\frac{\sigma_{st}(i)}{\sigma_{st}} $$

where *σ*_*st*_ is the number of shortest paths between nodes *s* and *t*, and *σ*_*st*_(*i*) is the number of those paths that go through node *i* (Opsahl et al. 2010).

The sum of the weights of edges of a node is called its strength or weighted degree (Barrat et al. 2004). By weighting degree based on capacity of incident links, major intersections of a city can be distinguished.

Alpha weighted degree (${C}_D^{w\alpha}(i)$) has been developed to evaluate the centrality of a node based on the number and the importance of its neighbors (Opsahl et al. 2010):

2 $$ {C}_D^{w\alpha}(i)={d}_i^{\left(i-\alpha \right)}\times {s}_i^{\alpha } $$

Here *S*_*i*_ is the flow entering node *i* from its incident links and *α* is a positive parameter. We assumed *α* to be equal to 0.5 which involves degree and strength to the same extent.

### Network abstraction

We studied networks of Chicago, Philadelphia, Anaheim, Birmingham, Gold Coast, Berlin (central district), and Isfahan (Iran). These cities are located in different areas of the world with different urban and social structures and different network sizes. We adopted the primal approach in abstracting the networks i.e. roads are abstracted as links and intersections are abstracted as nodes (Porta et al. 2006a, b). The direction of each link is determined following the direction of its corresponding road in the real network. For one-way streets, a directed link connects the tail node to the head. For two-way streets, two directed links are assumed to represent both directions of the street. We did not consider any weight for links.

The sizes of the networks are presented in Table 1.

**Table 1:** Size of the studied networks

City	Country	Number of Nodes	Number of Edges
Anaheim	USA	416	914
Austin	USA	6121	13,289
Birmingham	UK	12,300	28,059
Berlin	Germany	12,100	19,570
Chicago	USA	11,201	35,367
Gold coat	USA	4783	11,140
Isfahan	Iran	2150	4760
Philadelphia	USA	8202	20,467

## Results and discussion

**Fig. 1:**
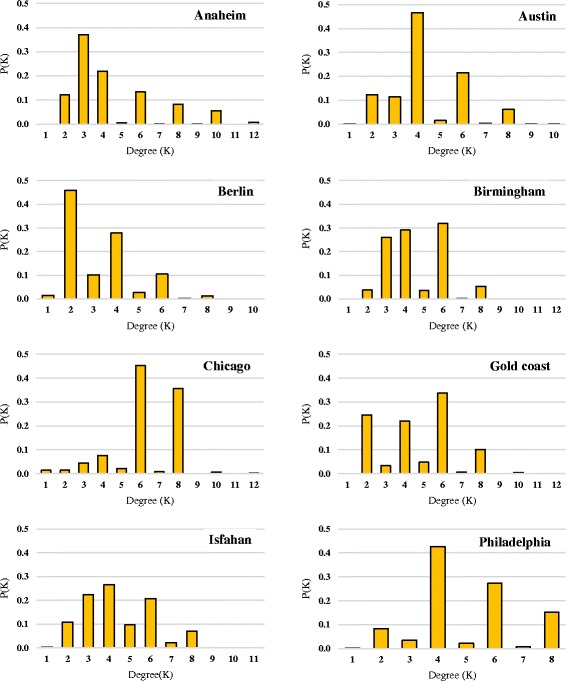
Degree distribution of studied urban road networks

Figure 1 shows the distribution of degrees of eight urban road networks under study. Limited variety of degree values is evident in the plots. Since the networks are directed, the node degrees assume odd and even values.

Except for being unimodal, the values of degrees do not show any similar order or distribution among cities. Moreover, it turns out that no power law distribution is observed in these networks. Therefore, it is established that for this sample of cities, no universal pattern could be derived from the degree distribution of urban road networks.

**Fig. 2:**
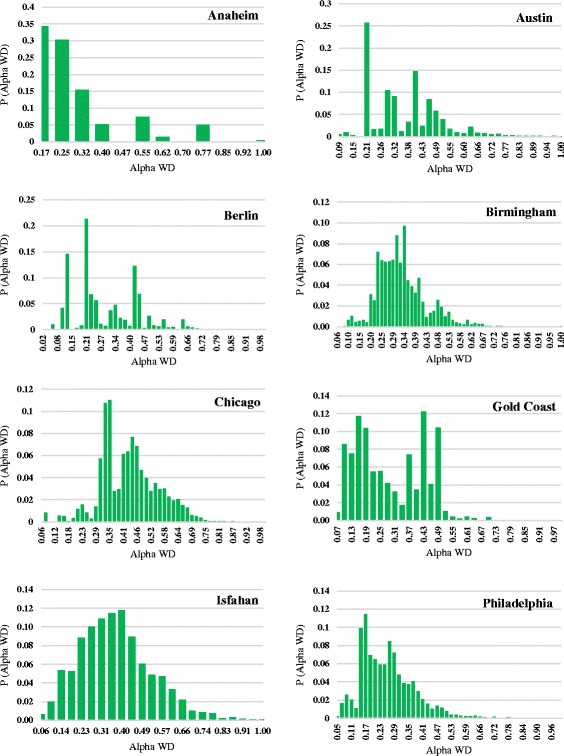
Alpha weighted degree distributions of eight urban transportation networks

Figure 2 shows the distribution of alpha weighted degrees of urban road networks. These distributions are even more diverse than degree distributions. Again, nothing general and similar to power law is observed although Anaheim shows a decreasing pattern of probability.

**Fig. 3:**
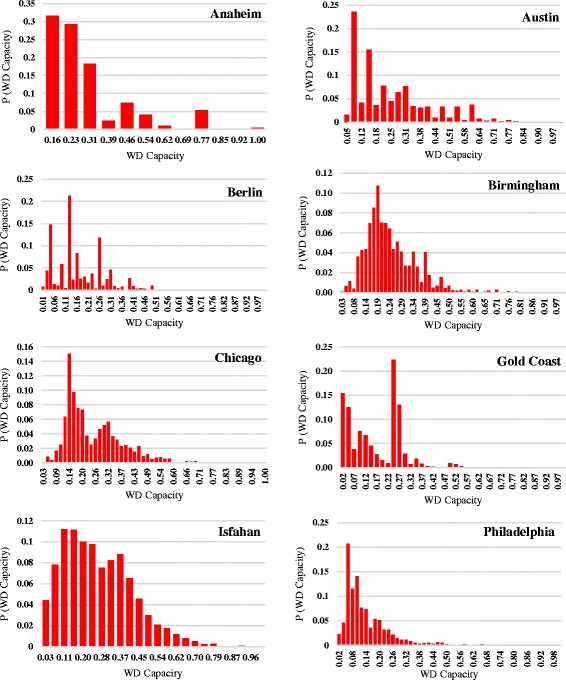
Capacity weighted degree distribution of eight urban transportation networks

Figure 3 shows the distribution of capacity weighted degree of studied urban road networks. Philadelphia shows a decreasing probability pattern but distributions are far from anything similar to scale free.

**Fig. 4:**
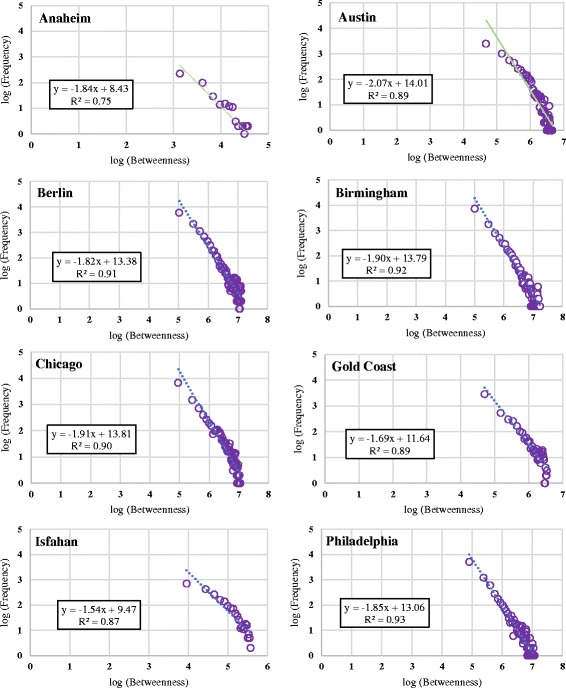
Log-log betweenness distribution of eight urban road networks

Figure 4 shows the log-log distribution of the betweenness centrality values of the nodes of urban road networks under study. Plots show a power law distribution. Inset equations show the determination coefficient (*R*2) and the power (as the coefficient of *x*). The power varies between 1.54 and 2.07. Coefficients of determination are all above 0.75.

These figures show that unlike other investigated centrality measures, betweenness centrality follows power law and urban road networks are scale free in terms of the betweenness centrality of their nodes. This result is interesting since we have not selected any specific distribution for degrees. Goh et al. (2001) showed that networks with power law distribution of degrees show power law distribution of betweenness centrality. For urban road networks, we show that regardless of degree distribution, betweenness centrality shows a power law distribution.

A question now arises about the susceptibility of the network to loss of its central nodes. This is investigated in the following subsection.

Cities are networks that gradually evolve. At the beginning, cities consisted of a main street and minor streets connecting to it. In these networks, nodes located on the main corridor had high betweenness value and their failure would cause a breakdown in the whole network. Gradually, loops started to form in the urban networks. Nodes on the peripheries would attain low betweenness and moreover, redundancy would keep the network from breakdown even if the highest central nodes failed. This is of course a conjecture. We’ll need to work on it in a different research.

**Fig. 5:**
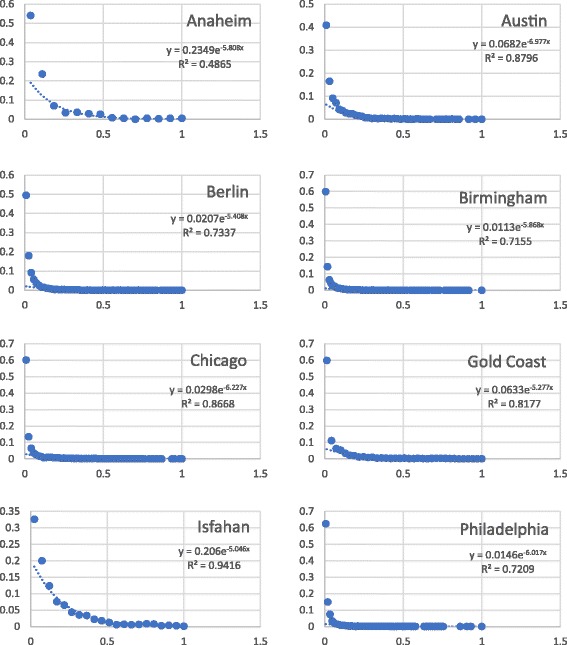
The exponential fitting of the data

The scattering of BC values may suggest an exponential distribution. We fitted exponential distribution to the data and show the results in Fig. 5.

It is evident that the coefficient of determination (R^2^) is higher in the case of power law indicating that the data is more similar to power distribution than exponential distribution.

### Vulnerability assessment

We adopted the node removal strategy to assess the criticality of nodes with high centrality values. We used the relative size of the giant component (RSGC) to measure the amount of damage to the network. For each urban network, we ranked nodes based on each centrality measure and carried out the node removal procedure in each network based on each four indices. Removals were carried out successively i.e. we did not update the centrality values at each removal step. Results are presented in Fig. 5. It is evident from these figures that nodes with highest values of betweenness are not always the most critical nodes. In Birmingham, after 10% of nodes are removed, and in Isfahan and Chicago, after 25% of nodes are removed, BC starts showing the most critical nodes. In other networks, BC does not show any specific advantage over other measures.

Therefore, we conclude that although the distribution of betweenness centrality values of urban road networks demonstrate power law behavior, this does not mean that nodes with highest values of BC are the most critical nodes. In other words, studied networks are not similar in the criterion for critical nodes. On the other hand, network under study are similar in their pattern of connectivity loss as they all show inverse-sigmoid like behavior. They all show a plateau for minor node removals followed by a sharp drop for removals of 5 to 30% node removals and converge to zero as the node removal percentage goes beyond 50%.

The fact that betweenness centralities follow power law but are not necessarily the most critical nodes of urban networks implies that the concept of betweenness centrality in urban road networks is more of functional merit than topological merit. This means that central nodes play an important role in transmitting the flow (functional merit) but their loss would not harm the network connectivity (topological merit). This could be due to the path redundancy in urban road networks.

**Fig. 6:**
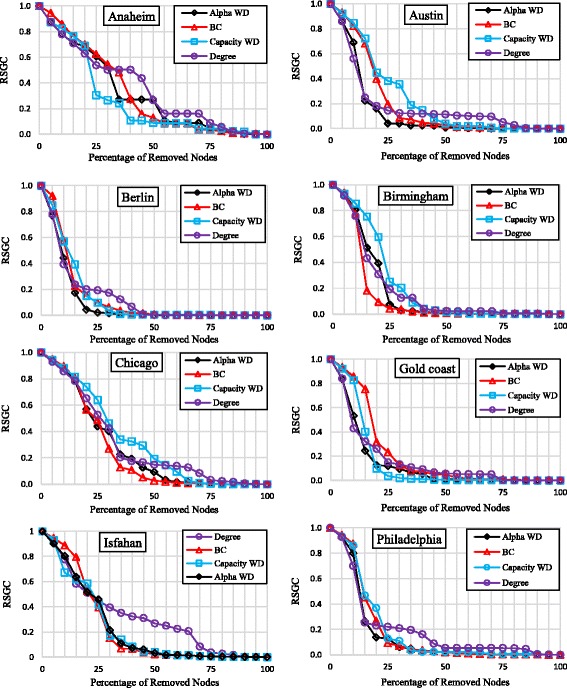
The change of RSGC against node removals

To support our claim, we analyze the correlation among node flow and node betweenness in Isfahan and Anaheim. These cities were selected because of flow data availability. Figure 6 shows these plots.

**Fig. 7:**
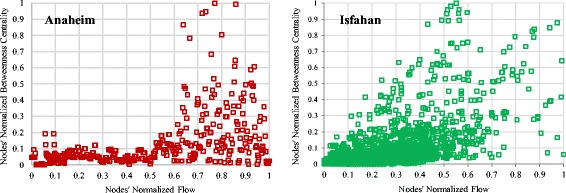
The correlation of normalized values of node flow and betweenness centrality

Figure 7 shows that nodes having low values of flow have low values of betweenness centrality but nodes with high values of flow, may have any value of betweenness.

## Conclusion

We investigated four node centrality measures including degree, betweenness centrality, capacity weighted degree, and alpha weighted degree for eight urban road networks. The planarity and spatial embeddedness of urban road networks limits the range of node degrees and prevents pursuing scale free characteristic based on degree distribution. Our results show that regardless of their degree distributions, betweenness centrality of urban network nodes show power law behavior. This is a more general observation compared to what Goh et al. (2001) have reported. We also showed that removing nodes with highest betweenness centrality values does not always significantly diminish the connectivity of networks.

We then analyzed the flow-betweenness correlation of two urban networks and observed that nodes with low flow necessarily have low centrality but nodes with high flow may or may not have high centrality.

Adding these two statements, we may conclude that in urban road networks, betweenness centrality is an important aspect from traffic point of view but not as important in topological point of view. This may be due the high redundancy of urban road networks. Nevertheless, power-law in the distribution of betweenness values indicate that there are few nodes that affect many trips. Detecting these nodes and securing the traffic flow passing through them would enhance the resilience of urban road networks. Providing redundancy by planning parallel corridors which in case of failure can carry the flow of vital nodes is one example of the actions that can be taken for securing the traffic flow passing through vital nodes. Another example is adopting traffic signal priority strategies. These strategies assign green time to approaches of neighboring intersections of a vital node such that no spill back forms in it. Spill back in vital nodes causes extra delay and possibly gridlock which makes the intersection breakdown. Securing the smooth traffic at these nodes by proper traffic signal priority and other traffic control strategies could assure the fluent traffic flow for a large portion of travelers.
